# The Efficacy of Misoprostol Vaginal Inserts for Induction of Labor in Women with Very Unfavorable Cervices

**DOI:** 10.3390/jcm12124106

**Published:** 2023-06-17

**Authors:** Maciej W. Socha, Wojciech Flis, Mateusz Wartęga, Martyna Stankiewicz, Aleksandra Kunicka

**Affiliations:** 1 Department of Perinatology, Gynecology and Gynecologic Oncology, Faculty of Health Sciences, Collegium Medicum in Bydgoszcz, Nicolaus Copernicus University, Łukasiewicza 1, 85-821 Bydgoszcz, Poland; 2 Department of Obstetrics and Gynecology, St. Adalbert’s Hospital in Gdańsk, Copernicus Healthcare Entity, Jana Pawła II 50, 80-462 Gdańsk, Poland; 3 Department of Pathophysiology, Faculty of Pharmacy, Collegium Medicum in Bydgoszcz, Nicolaus Copernicus University, M. Curie-Skłodowskiej 9, 85-094 Bydgoszcz, Poland

**Keywords:** labor induction, perinatology, cervical ripening, delivery

## Abstract

Background: The purpose of the present study was to evaluate the effectiveness of a misoprostol vaginal insert as an induction-of-labor (IOL) agent in women with an unfavorable cervix (Bishop score < 2) in achieving vaginal delivery (VD) within 48 h, depending on the gestational week, with particular emphasis on the cesarean section (CS) percentage, intrapartum analgesia application and possible side effects, such as tachysystole ratio. Methods: In this retrospective observational study involving 6000 screened pregnant patients, 190 women (3%) fulfilled the study inclusion criteria and underwent vaginal misoprostol IOL. The pregnant women were collected into three groups: patients who delivered at up to 37 weeks of gestation (<37 Group)—42 patients; patients who delivered between 37 and 41 weeks of gestation (37–41 Group)—76 patients; and patients who delivered after 41 weeks of gestation (41+ Group)—72 patients. The outcomes included time to delivery and mode of delivery, rate of tachysystole, need for intrapartum analgesia, and need for oxytocin augmentation. Results: Most of the patients delivered vaginally (54.8% in <37 Group vs. 57.9% in 37–41 Group vs. 61.1% in 41+ Group). A total of 89.5% (170/190) of patients delivered within 48 h (<37 Group—78.6% vs. 37–41 Group—89.5% vs. 41+ Group—95.8%). Statistical significance was demonstrated for the increased rate of vaginal deliveries and shortened time to delivery in the 41+ weeks group (*p* = 0.0026 and *p* = 0.0038). The indications for cesarean section were as follows: abnormal CTG pattern vs. lack of labor progression: 42.1% vs. 57.9% in <37 Group, 59.4% vs. 40.6% in 37–41 Group and 71.4% vs. 28.6% in 41+ Group. Statistical significance was demonstrated for the increased rate of abnormal CTG patterns as cesarean section indications in the 41+ Group (*p* = 0.0019). The need for oxytocin augmentation in each group was: 35.7% in <37 Group vs. 19.7% in 37–41 Group vs. 11.1% in 41+ Group. Statistical significance was shown for decreased need for oxytocin augmentation in +41 Group (*p* = 0.0016). The need for intrapartum anesthesia, depending on the group, was: 78.6% in <37 Group vs. 82.9% in 37–41 Group vs. 83.3% in 41+ Group. Statistical significance was demonstrated for increased need for intrapartum anesthesia application during labor in +41 Group (*p* = 0.0018). The prevalence of hyperstimulation was similar in all three groups (4.8% vs. 7.9% vs. 5.6% *p* > 0.05). Conclusions: The misoprostol vaginal regimen for IOL used in our study is effective in achieving vaginal delivery within 48 h. In post-term women, the use of this regimen is characterized by an increased rate of vaginal deliveries, a shorter time to delivery and a lower need for oxytocin.

## 1. Introduction

Labor induction remains the most widely used medical procedure in obstetrics worldwide. Over the years, labor induction strategies have been developed to most effectively influence the development of normal labor. Induction of labor (IOL) is the process of intentionally initiating contractions in pregnant patients who are not in labor to help them achieve vaginal delivery within 24–48 h. More than 20% of pregnant women undergo labor induction [1,2]. IOL is carried out for both fetal and maternal indications. Intravenous oxytocin infusion became a gold standard for achieving uterine contractions and remains extremely effective unless the cervix is unripe [3]. However, more than half of the women who undergo labor induction require prior cervical ripening. Therefore, most commonly, the labor induction protocol involves a cervical ripening agent followed by oxytocin/amniotomy as the next step. Prostaglandins (dinoprostone and misoprostol), progesterone antagonists, mechanical devices and other methods have been used to ripen the cervix with different efficacy and safety profile rates. From the point of view of successful labor induction, it is important to perform IOL quickly and effectively. An additional challenge is the successful induction of labor in women with a completely immature cervix.

Misoprostol is a synthetic prostaglandin E1 analog used for a variety of indications in the practice of obstetrics and gynecology, including medication abortion, induction of labor, cervical ripening before surgical procedures and the treatment of postpartum hemorrhage [4]. Misoprostol’s effects are dose-dependent and include cervical softening and dilation. Additionally, misoprostol has the ability to directly induce uterine contractions by contracting smooth muscle fibers in the myometrium [5,6]. Misoprostol can be administered vaginally or orally as a cervical ripening agent [7]. Prostaglandin analogs (misoprostol and dinoprostone) are considered the most effective ripening agents for women with an unfavorable cervix [4,7,8]. In addition, these substances are also effective in IOL. However, their main side effect is uterine tachysystole with or without cardiotocographic abnormalities. These effects are route- and dose-dependent [9,10].

The purpose of this study was to evaluate the effectiveness of the misoprostol vaginal insert in achieving vaginal delivery in women with an unfavorable cervix depending on the gestational week, with additional emphasis on the safety profile and possible side effects, such as tachysystole ratio.

## 2. Materials and Methods

This was a retrospective, observational, single-center cohort study. Consent for the analysis was given by the Institutional Ethics Committee of Collegium Medicum in Bydgoszcz, Nicolaus Copernicus University. The study was conducted in accordance with the Declaration of Helsinki.

The analysis included the medical records of 6000 pregnant patients who underwent IOL at the Department of Obstetrics and Gynecology, St. Adalbert’s Hospital in Gdańsk (tertiary referral hospital) between 1 January 2018 and 31 December 2020. The data were generated on the basis of electronic medical records collected by medical staff. In addition, the dataset was checked for possible errors, and any detected inconsistencies were verified. Data on the woman in labor, the course of childbirth and the condition of the newborn were recorded in a computer database by midwives and doctors during and immediately after labor. After a thorough analysis of the medical documentation, taking into account all the criteria described, the following information was obtained: obstetric history, duration of pregnancy (determined on the basis of the date of the last menstruation, confirmed by the first trimester USG), type of cervical ripening agent used, course and complications of the current pregnancy, course of the IOL, duration of labor, and patient demographic data. Indications for IOL were in accordance with the current recommendations of the Polish Society of Gynecologists and Obstetricians [11]. Informed consent was obtained from all participants before the start of the induction procedure.

IOL consisted of 200-microgram misoprostol vaginal inserts in all included patients. In the absence of initiated labor, defined as the occurrence of regular systolic activity within 24 h, oxytocin was used in the local protocol for induction of labor. During labor, the patients were given intrapartum analgesia in the form of an intravenous infusion of Remifentanil on demand.

The study included patients who qualified for induction of labor for both maternal and fetal indications and with a completely unfavorable cervix (Bishop score < 2) for economic reasons. All patients were adults. The patients were in full-term pregnancy (>37 weeks), preterm pregnancy (below 37 weeks) or post-term pregnancy (>41 weeks).

**Figure 1:**
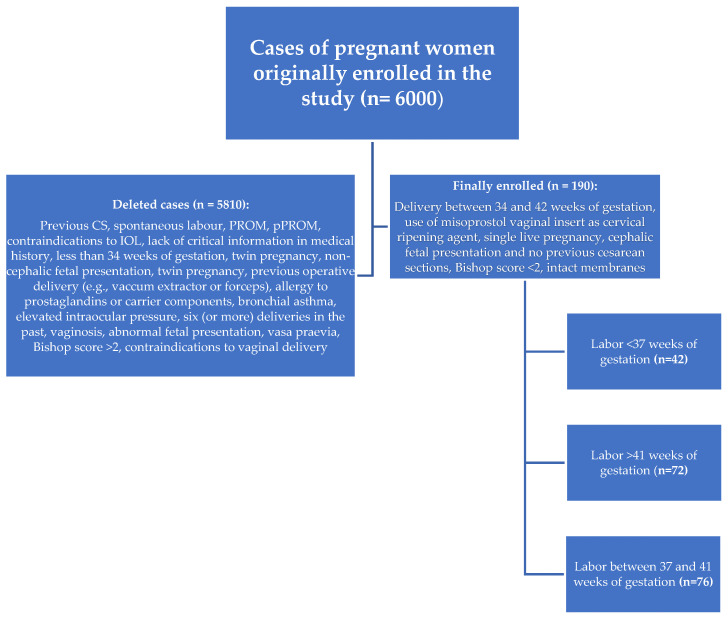
Inclusion and exclusion criteria for the study population.

The criteria for inclusion in the study group were: delivery between 34 and 42 weeks of gestation, use of a misoprostol vaginal insert as a cervical ripening agent, single live pregnancy, cephalic fetal presentation, no previous cesarean sections, intact membranes and a Bishop score < 2. Selected patients showed no signs of labor until IOL began. The exclusion criteria were: the onset of labor, previous cesarean section, premature rupture of membranes (PROM and pPROM), twin pregnancy, previous operative delivery (e.g., vacuum extractor or forceps), allergy to prostaglandins or carrier components, bronchial asthma, elevated intraocular pressure, six (or more) deliveries in the past, gestational age less than 34 weeks of gestation, vaginosis, abnormal fetal presentation, vasa praevia, a Bishop score > 2, and any contraindications to vaginal delivery and IOL in accordance with the Polish guidelines. An analysis of the documentation covering 6000 deliveries at the analyzed time was carried out, of which, based on the adopted criteria, 190 cases qualified for further analysis (Figure 1). Indications for labor induction were mainly: large for gestational age (LGA), small for gestational age (SGA), fetal growth restriction (FGR), oligohydramnios, polyhydramnios, gestational diabetes mellitus (GDM) type 1 or 2, pregnancy-induced hypertension (PIH), preeclampsia (PE), and post-term pregnancy.

The primary outcome was an evaluation of the effectiveness of vaginal misoprostol in achieving delivery (vaginal or by cesarean section) within 48 h, with an emphasis on women who delivered within 24 h. The secondary outcomes were the proportion of women who underwent a cesarean section and oxytocin administration and for whom the application of intrapartum analgesia was necessary. We assessed the effectiveness of triggering uterine contraction activity after the administration of a vaginal insert with misoprostol. We compared the groups in terms of the percentage of vaginal births (VBs), the percentage of cesarean sections (CSs) and the most common indications for these procedures. We also compared the groups in terms of the presence of uterine hyperstimulation (tachysystole) and the need for intravenous intrapartum analgesia. Tachysystole was diagnosed based on the following criteria: contractions lasting > 2 min, no significant interval between contractions and >5 contractions in 10 min. In case of tachysystole, an intravenous infusion of 25 micrograms of Fenoterol was used.

## 3. Statistical Analysis

The statistical analysis was performed in Gdańsk, Poland using the statistical suite StatSoft—Inc. (2014)—STATISTICA version 12.0 (a data analysis software system) and Microsoft Excel version 2305.

The quantitative variables were characterized by the arithmetic means of standard deviations or medians or maxima/minima (ranges) and 95% confidence intervals. The qualitative variables were presented with the use of counts and percentages. In order to check if a quantitative variable derived from a population with a normal distribution, the W Shapiro–Wilk test was used, whereas to prove the hypotheses on the homogeneity of variances the Leven (Brown–Forsythe) test was utilized. The significance of the difference between more than two groups was assessed with the F test (ANOVA) or the Kruskal–Wallis test. In the case of statistically significant differences between two groups, post hoc tests were utilized (Tukey’s test for F or Dunn’s test for Kruskal–Wallis). The significance of the difference between more than two variables in the paired variables model was checked by analysis of variance with repeated measurements or Friedman’s test. Chi-squared tests for independence were used for qualitative variables. In order to determine the dependence, strength and direction between variables, correlation analysis was used by determining Pearson’s or Spearman’s correlation coefficients. In all the calculations, the statistical significance level of *p* = 0.05 was used.

## 4. Results

A total of 190 pregnant women (34 to 42 weeks of gestation) were included in the study. The flow chart for patient selection is shown in Figure 1. Then, 190 previously selected patients were divided into three groups: patients who delivered at up to 37 weeks of gestation (preterm birth) (<37 Group); patients who delivered between 37 and 41 weeks of gestation (37-41 Group); and patients who delivered after 41 weeks of gestation (41+ Group). The ages of the patients ranged from 19 to 37 years, with a median age of 25 years. A total of 112 patients (58%) were primiparous, and the remaining 78 (42%) were multiparous.

The mean gestational age in <37 Group was 35.1 (0.7) (ranging from 34.0 to 36.0), in 37–41 Group it was 38.5 (1.2) (ranging from 37.0 to 40.0) and in +41 Group it was 41.1 (0.3) (ranging from 41.0 to 42.0) (Table 1).

Most patients delivered within 48 h (vaginally or by cesarean section)—89.5% (170/190). Of the total of 42 women in <37 Group, 78.6% had times to delivery of up to 48h and 21.4% had times to delivery >48 h; in 37–41 Group, the corresponding figures were 89.5% and 10.5%; and in +41 Group, these figures were 95.8% and 4.2%. Additionally, the time to delivery (vaginal or cesarean) was statistically significantly shorter in +41 Group in comparison to <37 Group (*p* = 0.0038). No statistically significant differences were found for the others.

Most patients had vaginal delivery in each study group after misoprostol administration. There were 23 vaginal deliveries (54.8%) in <37 Group and 44 (57.9%) in 37–41 Group as compared to 44 (61.1%) in +41 Group. The cesarean section rate was 45.2% (<37 Group) vs. 42.1% (37–41 Group) vs. 38.9% (+41 Group). The percentage of vaginal delivery was statistically significantly higher in +41 Group than in <37 Group (*p* = 0.0026) (Table 1).

Of the 23 women who had a vaginal delivery (VD) in <37 Group, 87.0% had a time to vaginal delivery of up to 48 h and 13.0% had a time >48 h. In 37–41 Group, these figures were 95.5% and 4.5%. Surprisingly, 93.2% of women in 37–41 Group had VDs within 24 h. All patients who had vaginal delivery in +41 Group delivered within 48 h, of which as many as 97.7% had parturition within 24 h. The time to vaginal delivery was statistically significantly shorter in +41 Group in comparison to <37 Group (*p* = 0.0142) (Table 2).

Of the 19 women in <37 Group who delivered by CS, 68.4% had a time for cesarean delivery of up to 48 h and 31.6% had a time >48 h. In 37–41 Group, the corresponding figures were 81.2% and 18.8%. Finally, in +41 Group, these figures were 89.3% and 10.7%. Additionally, almost 75% of patients had CSs up to 24 h. The time to cesarean delivery was statistically significantly shorter in +41 Group in comparison to <37 Group (*p* < 0.0001)**.** No statistically significant differences were found for the other comparisons (Table 3).

The distribution of the indications for cesarean section was as follows: in <37 Group, the dominant indication for cesarean section was the lack of progress in labor (57.9%), while in 37–41 Group and +41 Group, an abnormal CTG record was dominant (59.4% and 71.4%). Abnormal CTG record as an indication for CS was encountered statistically significantly more often in +41 Group than in <37 Group (*p* = 0.0019), regardless of the time to delivery. The lack of labor progression occurred statistically significantly less often in +41 Group in comparison to <37 Group (*p* = 0.0019) (Table 4 and Table 5).

A total of 35.7% of patients in <37 Group required oxytocin IOL, while in 37–41 Group the corresponding figure was 19.7% and in +41 Group it was only 11.1%. The least demand for oxytocin was exhibited by +41 Group. The need for oxytocin administration was statistically significantly more often encountered in <37 Group in comparison with +41 Group (*p* = 0.0016) (Table 6).

A total of 78.6% of women in <37 Group requested intrapartum analgesia. In 37–41 Group, the corresponding figure was 82.9%, and in +41 Group it was 83.3%. Considering the time to delivery and the use of analgesics, in <37 Group, 75.8% of women who had delivery within 24 h required intravenous analgesia. In 37–41 Group, the corresponding figure was 88.9%. Finally, in +41 Group, it was 96.7%. The use of intrapartum analgesia was statistically significantly more often required in +41 Group in comparison with 37–41 Group in terms of delivery up to 24 h (*p* = 0.0018) (Table 6).

Uterine hyperstimulation occurrence was similar for all groups and remained low. There were no significant differences between the groups with respect to tachysystole (*p* > 0.05) (Table 7).

## 5. Discussion

As mentioned before, almost 20% of pregnant women undergo IOL, and in many cases patients present with a very unfavorable cervix, limiting clinical satisfaction results. To achieve a successful IOL, cervical ripening is a crucial initiation, and subsequent adequate and powerful uterine contractions at a regular frequency are required. Currently, there are generally two main strategies to achieve proper cervical ripening: mechanical (e.g., Foley or Cook catheter) and pharmacological methods, such as progesterone antagonists, nitric oxide donors and prostaglandins (PGs) [12,13,14]. Oxytocin is a decisive contraction factor. However, it has minimal effect on cervical ripening, and in patients with a very unfavorable cervix it might even lead to adverse outcomes. Therefore, misoprostol seems to be the perfect agent for IOL because it causes cervical ripening directly and actively affects the triggering of uterine contractions [4]. Therefore, misoprostol (a PGE1 analog) is commonly used as an IOL agent [7,15]. Although misoprostol is not licensed for labor induction, it is widely and successfully used off-label for IOL worldwide [16,17,18,19,20,21].

The aim of this study was to evaluate the efficacy of misoprostol vaginal inserts in achieving vaginal delivery in women with an unprepared cervix, defined as a Bishop score lower than 2 points.

An unfavorable or unripe cervix can be defined as one that has undergone minimal changes and is less vulnerable to attempts at labor induction. Our theoretical research has shown that misoprostol (affecting the relevant receptors and metabolic pathways) can cause significant changes in cervical tissue, leading to cervical ripening. Therefore, we decided to research cases of women with an immature cervix to evaluate the efficacy of misoprostol as an agent for cervical ripening and labor induction. We consider this a very interesting topic because there are currently few studies on the use of misoprostol in the exceedingly immature cervix. In addition, we managed to gather a fascinating group of patients for the study, which allowed us to consider both term and preterm deliveries.

Efficacy was demonstrated for individual groups because the vast majority of women delivered within 48 h, most of whom had vaginal parturition (VD). It is also worth noting that, when considering only vaginal deliveries, more than 90% of patients in 37-41 Group and +41 Group had vaginal delivery within 24 h (93.2% and 97.7%). These data are consistent with other research results [4,22]. Decreasing the time to delivery may have several benefits. Prolonged labor is associated with higher infection rates, greater use of antibiotics, increased maternal distress, more use of oxytocin, a greater percentage of instrumental deliveries, and more demands on staff and hospital resources [23]. Additionally, studies show that delivery time is highly important to women undergoing IOL. Moreover, women often perceive long-duration labor inductions as traumatic [24,25].

The primary outcome was an assessment of the efficacy of misoprostol IOL. Overall, the vast majority of patients had a vaginal delivery, and almost all of them (89.5%) delivered within 48 h. In the study population, the majority of patients (regardless of gestational age) had a successful vaginal delivery (54.8%, 57.9% and 61.1%), and a significant proportion of them delivered within 48 h (87%, 95.5% and 100%). Additionally, the groups of patients with full-term pregnancy (37–41 Group) and post-term pregnancy (+41 Group) were characterized by a high percentage of vaginal deliveries within 24 h. These data show that misoprostol may be a highly effective IOL agent. Despite the fact that these data are not statistically significant, they are consistent with the research conducted so far on this issue [2,26,27].

What is extremely interesting is that our study showed statistical significance in shortening the time to delivery (within 48h) and an increase in the percentage of vaginal deliveries in the group of patients with post-term pregnancy (*p* = 0.0038 and *p* = 0.0026, respectively) in comparison with preterm pregnancies considering patients with an unfavorable cervix (Bishop < 2). This is consistent with other studies which concluded that misoprostol-augmented IOL leads to an increase in vaginal delivery rate [28,29,30,31,32,33,34,35]. It is also worth noting that the vast majority of post-term pregnant women who had VDs delivered within 24 h. Again, these data are consistent with other studies conducted in this field [36].

Considering cesarean section, most women who underwent CS delivered within 48 h (68.4% vs. 81.2% vs. 89.3%). Interestingly, the time to cesarean delivery was statistically significantly shorter in the case of post-term pregnancies than in the case of preterm delivery. It is clear from the above data that the use of vaginal misoprostol in post-term pregnancy IOL resulted in an increase in the percentage of vaginal deliveries. In the case of cesarean delivery, the time to delivery was statistically significantly shortened in the group of post-term pregnancies. However, quite a few published studies contradict our results. A few authors have concluded that IOL with misoprostol strongly increases the risk of cesarean delivery and leads to prolonged labor [32,37,38,39]. The differences in results are likely due to differences in study designs. Most studies do not differentiate the effect of misoprostol based on gestational age but only consider its effectiveness in labor induction and maternal and fetal outcomes. Our study also has many limitations, such as the lack of a distinction between multiparous and primiparous women. In addition, we did not consider possible fetal complications and maternal factors, such as pregnancy complications (e.g., preeclampsia or gestational diabetes mellitus). Therefore, we believe these results can be a great starting point for further research in this field. In our study, the two most frequently occurring indications for CS were abnormal CTG recordings and lack of labor progression, regardless of patient parity. Therefore, our study further highlights the fact that it is crucial to monitor patients being induced with vaginal misoprostol in case of fetal heart-rate abnormalities. The study showed unequivocally that abnormal CTG recordings occurred statistically significantly more often in the group of patients with post-term pregnancy (+41 Group) than in the group with preterm delivery (<37 Group). Similarly, the lack of labor progression was statistically significantly less often encountered in +41 Group than in <37 Group (*p* = 0.0019). This result agrees with the studies of other authors who claim that misoprostol significantly increases the risk of fetal heart-rate disturbances during IOL with misoprostol [40,41,42]. We also believe that the increased rate of abnormal CTG recordings in post-term pregnancies may be due to the high maturity of the placenta and its tendency to fail at this stage of pregnancy [43].

We also noticed that the need for oxytocin IOL was statistically significantly more often required in <37 Group (preterm labor) compared to +41 Group (*p* = 0.0016). The above data may suggest that misoprostol in preterm labor reduced the potential for uterine contraction generation. We believe that a possible explanation of this phenomenon lies in the uterine muscle and its prostaglandin receptors. Misoprostol acts via four membrane-attached prostaglandin EP receptors (EP 1–EP 4). The EP1 and EP3 receptors facilitate smooth muscle contractility, while EP2 and EP4 promote smooth muscle relaxation. During pregnancy, the concentrations of prostaglandin receptors are downregulated to maintain uterine quiescence, with a significant increase in concentration at term. Therefore, we suggest that during preterm labor, the expression of prostaglandin receptors is not adequate to ensure the adequate generation of contractile activity by misoprostol [7,14]. The lowest need for subsequent oxytocin IOL characterized group 3. This may be due to the fact that, in postpartum pregnancy, the uterine muscle has the highest expression of prostaglandin receptors. Therefore, the use of misoprostol may be more effective in this group. However, this topic requires further research.

In each study group, most patients were willing to use intrapartum analgesia (IV Remifentanil). This result is in accordance with recent reports. According to Redling K., et al., patients receiving a vaginal insert with misoprostol more often required intrapartum analgesia with opioids [35]. However, due to the methodology of our study, these figures may be overestimated. The subjective and multidimensional nature of the pain experience renders pain assessment really challenging. The differences in the results may be because no cut-off threshold for analgesia was adopted in our study. In the absence of a defined pain scale for this study, the assessment of pain was very dependent on the individual predisposition of the patient and her pain threshold, which made it very subjective. As a result, patients received analgesia on a subjective basis.

Additionally, no objective pain rating scale (e.g., the Visual Analog Scale or the Numeric Rating Scale) was used in our study [44]. Therefore, it is impossible to assess the actual severity of pain objectively. We believe this area deserves a more thorough investigation. However, interestingly, the use of intrapartum analgesia was statistically significantly more frequent in +41 Group than in 37–41 Group in terms of delivery within 24 h. These data may suggest that more rapid progress of labor (in the group of post-term pregnancies) may be associated with an increased demand for intravenous analgesia during labor. However, it should be remembered that intravenous anesthesia (Remifentanil) is off-label. A recent study showed that, although intravenous anesthesia provides a satisfactory experience, the use of epidural analgesia provides better analgesia and satisfaction than patient-controlled analgesia with Remifentanil [45]. Additionally, recently, a high proportion of Polish women chose epidural analgesia during labor over other pharmacological and non-pharmacological pain-relief methods [46]. The reason why Polish patients tend to have an increased demand for choosing epidural or intravenous analgesia might be a lack of proper counseling about the possible non-pharmacological methods of pain relief during labor given by healthcare professionals. Women have insufficient information about non-pharmacological methods of pain relief. Non-pharmacological pain-relief techniques include transcutaneous electrical nerve stimulation (TENS), water immersion, aromatherapy, acupuncture, and acupressure and massage techniques [47,48,49]. In many cases, information about possible methods of intrapartum analgesia application is given exclusively at the beginning of labor. We believe it is critical to increase patients’ knowledge about non-pharmacological analgesia methods, which may lead to wider use of non-pharmacological pain-relief methods and general improvements in women’s birth experiences. Considering the above, we believe that the conduction of additional studies on using vaginal misoprostol and epidural anesthesia is necessary to evaluate their effectiveness. Interestingly, acetaminophen, as a non-opioid intrapartum analgesia, has recently gained more attention. According to the studies that have been carried out, intravenous acetaminophen is an efficacious non-opioid drug for relieving labor pain without significant maternal or fetal adverse effects [50].

Hyperstimulation occurred in each group with a similar frequency, i.e., in a small percentage of patients. Among all 190 women enrolled in the study, tachysystole occurred in only 12 women (6.3%). With such a low percentage, the amount of hyperstimulation is negligible. The rate of uterine hyperstimulation was not statistically significant. However, this result may be underestimated. In medical records, the indication for cesarean section is often an abnormal CTG recording, which may accompany or result from hyperstimulation which was not recognized in favor of a diagnosis of impending fetal asphyxia. The presented data suggest that vaginal misoprostol has a low complication rate. However, on the other hand, studies show that the use of vaginal misoprostol may be associated with an increased percentage of uterine hyperstimulation [51,52,53]. Additionally, the differences in the results may probably be due to the different designs of the studies and the different ways of dosing vaginal misoprostol. Therefore, we believe that this topic requires further, thorough research.

Our study has its limitations, such as the lack of division of patients into primiparous and multiparous women and the lack of assessment of patients in terms of their body mass indexes (BMIs). In addition, no clinical scale was used to assess pain. Therefore, we believe it is crucial to conduct additional studies that will assess the effectiveness of misoprostol in more detail, considering additional variables.

Our retrospective study enhances our understanding of using vaginal misoprostol for IOL in current obstetrical practice. The inclusion criteria meant that a wide range of women were eligible for labor induction with misoprostol. Nevertheless, due to the retrospective nature of our study, the results should be interpreted cautiously, as there was no control group. Nevertheless, our results provide evidence for the use of misoprostol, showing that most patients with an unfavorable cervix achieved successful labor within 48 h (vaginally or by cesarean section). At the same time, it has been shown that its use significantly increased the percentage of vaginal deliveries in the group of post-term pregnant women and shortened the duration of labor. This is particularly relevant, as there were medical reasons why, in this group of women, continuing pregnancy was potentially unsafe for either the mother or fetus.

## 6. Conclusions

Vaginal misoprostol is an effective cervical ripening agent and is effective in achieving vaginal delivery, especially in women with post-term pregnancies with a very unfavorable cervix.

- The use of vaginal misoprostol in labor induction is highly effective in achieving vaginal delivery within 48 h in women with a very unfavorable cervix (Bishop < 2).
- The use of misoprostol may be associated with shortening the time to delivery (both vaginal and cesarean).
- The use of misoprostol in a group of post-term pregnancies with very unfavorable cervices significantly increased the rate of vaginal deliveries and allowed successful vaginal delivery within 24 h.
- The use of misoprostol in a post-term pregnancy group with very unfavorable cervices was associated with a lower need for oxytocin augmentation.
- Misoprostol vaginal administration for IOL may be associated with an increased desire to use analgesia during labor.
- The use of vaginal misoprostol in post-term pregnant patients with a very unfavorable cervix is associated with an increased risk of abnormal CTG recording as an indication for CS and may be associated with a lower risk of labor arrest.
- Induction of labor with misoprostol in preterm labor may require more frequent use of oxytocin augmentation.
- A misoprostol IOL regimen might be considered a safe IOL agent due to the small percentage of tachysystole cases that occurred.

## Figures and Tables

**Table 1:** Comparative analysis of the three groups—<37 Group, 37–41 Group and +41 Group—with respect to gestational age, time to labor and parturition type.

	<37 Group (*n* = 42)	37–41 Group (*n* = 76)	+41 Group (*n* = 72)	All (*n* = 190)	*p*-Value
**Gestational age**					
Mean (SD)	35.1 (0.7)	38.5 (1.2)	41.1 (0.3)	38.8 (2.4)	^a–c^ <0.0001 ^3^
Range	34.0–36.0	37.0–40.0	41.0–42.0	34.0–42.0	
Median	35.0 ^a,b^	38.0 ^a,c^	41.0 ^b,c^	40.0	
95% CI	[34.9; 35.4]	[38.2; 38.8]	[41.1; 41.2]	[38.4; 39.1]	
**Time to delivery**	a	b	c		
24 h	27 (64.3%)	61 (80.3%)	64 (88.9%)	152 (80.0%)	^a,b^ 0.1064 ^4^
24–48 h	6 (14.3%)	7 (9.2%)	5 (6.9%)	18 (9.5%)	^a–c^ 0.0038 ^1,4^
Up to 48 h	33 (78.6%)	68 (89.5%)	69 (95.8%)	170 (89.5%)	^b,c^ 0.7971 ^4^
>48 h	9 (21.4%)	8 (10.5%)	3 (4.2%)	20 (10.5%)	
**Delivery type**	a	b	c		^a,b^ 0.7422 ^4^
Vaginal	23 (54.8%)	44 (57.9%)	44 (61.1%)	111 (58.4%)	^a–c^ 0.0016 ^4^
Cesarean section (CS)	19 (45.2%)	32 (42.1%)	28 (38.9%)	79 (41.6%)	^b,c^ 0.6904 ^4^

^1^ ANOVA. ^2^ Kruskal–Wallis. ^3^ Post hoc. ^4^ Chi-square; ^a^—<37 Group; ^b^—37-41 Group; ^c^—+41 Group.

**Table 2:** Comparative analysis of the three groups—<37 Group, 37–41 Group and +41 Group—with respect to time to delivery for vaginal delivery.

	<37 Group (*n* = 23)	37–41 Group (*n* = 44)	+41 Group (*n* = 44)	All (*n* = 111)	*p*-Value
**Time to vaginal delivery**	a	b	c		^a,b^ 0.2088 ^4,3^
24 h	15 (65.3%)	41 (93.2%)	43 (97.7%)	99 (89.2%)	^a–c^ 0.0142 ^1,4,^
24–48 h	5 (21.7%)	1 (2.3%)	1 (2.3%)	7 (6.3%)	^b,c^ 0.1526 ^2,4^
up to 48 h	20 (87.0%)	42 (95.5%)	44 (100.0%)	106 (95.5%)	
>48 h	3 (13.0%)	2 (4.5%)	0 (0.0%)	5 (4.5%)	

^1^ ANOVA. ^2^ Kruskal–Wallis. ^3^ Post hoc. ^4^ Chi-square; ^a^—<37 Group; ^b^—37–41 Group; ^c^—+41 Group.

**Table 3:** Comparative analysis of the three groups—<37 Group, 37–41 Group and +41 Group—with respect to time to delivery by cesarean section.

	<37 Group (*n* = 19)	37–41 Group (*n* = 32)	+41 Group (*n* = 28)	All (*n* = 79)	*p*-Value
**Time to delivery by cesarean section**	a	b	c		^a,b^ 0.2964 ^4,3^
24 h	12 (63.2%)	20 (62.4%)	21 (75.0%)	53 (67.1%)	^a–c^ <0.0001 ^1,4^
24–48 h	1 (5.2%)	6 (18.8%)	4 (14.3%)	11 (13.9%)	^b,c^ 0.3845 ^2,4^
up to 48 h	13 (68.4%)	26 (81.2%)	25 (89.3%)	64 (81.0%)	
>48 h	6 (31.6%)	6 (18.8%)	3 (10.7%)	15 (19.0%)	

^1^ ANOVA. ^2^ Kruskal–Wallis. ^3^ Post hoc. ^4^ Chi-square; ^a^—<37 Group; ^b^—37-41 Group; ^c^—+41 Group.

**Table 4:** Comparative analysis of the three groups—<37 Group, 37–41 Group and +41 Group—with respect to abnormal CTG records as indications for cesarean delivery.

	<37 Group (*n* = 19)	37–41 Group (*n* = 32)	+41 Group (*n* = 28)	All (*n* = 79)	*p*-Value
**Abnormal CTG record**	a	b	c		^a,b^ 0.2322 ^4,1^
TOTAL	8 (42.1%)	19 (59.4%)	20 (71.4%)	47 (59.5%)	^a–c^ 0.0019 ^2,4^
					^b,c^ 0.5121 ^4^
24 h	7 (87.5%)	16 (84.2%)	15 (75.0%)	38 (80.8%)	^a,b^ 0.2322 ^3,4^
24–48 h	0 (0.0%)	2 (10.5%)	4 (20.0%)	6 (12.8%)	^a–c^ 0.4863 ^4^
up to 48 h	7 (87.5%)	18 (94.7%)	19 (95.0%)	44 (93.6%)	^b,c^ 0.9703 ^4^
>48 h	1 (12.5%)	1 (5.3%)	1 (5.0%)	3 (6.4%)	

^1^ ANOVA. ^2^ Kruskal–Wallis. ^3^ Post hoc. ^4^ Chi-square; ^a^—<37 Group; ^b^—37–41 Group; ^c^—+41 Group.

**Table 5:** Comparative analysis of the three groups—<37 Group, 37–41 Group and +41 Group—with respect to failure to labor progress as an indication for cesarean section.

	<37 Group (*n* = 19)	37–41 Group (*n* = 32)	+41 Group (*n* = 28)	All (*n* = 79)	*p*-Value
**Failure to labor progression**	a	b	c		^a,b^ 0.2322 ^4,1^
TOTAL	11 (57.9%)	13 (40.6%)	8 (28.6%)	32 (40.5%)	^a–c^ 0.0019 ^2,4^
					^b,c^ 0.3288 ^4^
24 h	5 (45.5%)	4 (30.8%)	6 (75.0%)	15 (46.9%)	^a,b^ 0.72923 ^3,4^
24–48 h	1 (9.0%)	4 (30.8%)	0 (0.0%)	5 (15.6%)	^a–c^ 0.3615 ^4^
up to 48 h	6 (54.5%)	8 (61.6%)	6 (75.0%)	20 (62.5%)	^b,c^ 0.5251 ^4^
>48 h	5 (45.5%)	5 (38.4%)	2 (25.0%)	12 (37.5%)	

^1^ ANOVA. ^2^ Kruskal–Wallis. ^3^ Post hoc. ^4^ Chi-square; ^a^—<37 Group; ^b^—37–41 Group; ^c^—+41 Group.

**Table 6:** Comparative analysis of the three groups—<37 Group, 37–41 Group and +41 Group—with respect to oxytocin and analgesia use.

	<37 Group (*n* = 42)	37–41 Group (*n* = 76)	+41 Group (*n* = 72)	All (*n* = 190)	*p*-Value
**Oxytocin use**	a	b	c		^a,b^ 0.0563 ^4,1^
TOTAL	15 (35.7%)	15 (19.7%)	8 (11.1%)	38 (20.0%)	^a–c^ 0.0016 ^2,4^
					^b,c^ 0.1477 ^4^
	a	b	c		^a,b^ 0.7125 ^4^
24–48 h	6 (40.0%)	7 (46.7%)	5 (62.5%)	18 (47.4%)	^a–c^ 0.3036 ^3,4^
48 h	9 (60.0%)	8 (53.3%)	3 (37.5%)	20 (52.6%)	^b,c^ 0.4691 ^4^
**Intrapartum analgesia (Remifentanyl)**	a	b	c		^a,b^ 0.5637 ^4^
TOTAL	33 (78.6%)	63 (82.9%)	60 (83.3%)	156 (82.1%)	^a–c^ 0.5270 ^4^
					^b,c^ 0.9433 ^4^
	a	b	c		^a,b^0.0924 ^4^
24 h	25 (75.8%)	56 (88.9%)	58 (96.7%)	139 (89.1%)	^a–c^ 0.0018 ^4^
24–48 h	0 (0.0%)	0 (0.0%)	0 (0.0%)	0 (0.0%)	^b,c^ 0.0978 ^4^
>48 h	8 (24.2%)	7 (11.1%)	2 (3.3%)	17 (10.9%)	

^1^ ANOVA. ^2^ Kruskal–Wallis. ^3^ Post hoc. ^4^ Chi-square; ^a^—<37 Group; ^b^—37–41 Group; ^c^—+41 Group.

**Table 7:** Comparative analysis of the three groups—<37 Group, 37–41 Group and +41 Group—with respect to occurrence of tachysystole.

	<37 Group (*n* = 42)	37–41 Group (*n* = 76)	+41 Group (*n* = 72)	All (*n* = 190)	*p*-Value
**Occurrence of tachysystole**	a	b	c		^a,b^ 0.5169
TOTAL	2 (4.8%)	6 (7.9%)	4 (5.6%)	12 (6.3%)	^a–c^ 0.8548 ^4^
					^b,c^ 0.5709 ^4^

^4^ Chi-square; ^a^—<37 Group; ^b^—37–41 Group; ^c^—+41 Group.

## Data Availability

The data presented in this study are available on request from the corresponding author. The data are not publicly available due to privacy restrictions.

## References

[1] Nicholson J., Kellar L., Henning G., Waheed A., Colon-Gonzalez M., Ural S. (2015). The association between the regular use of preventive labour induction and improved term birth outcomes: Findings of a systematic review and meta-analysis. BJOG Int. J. Obstet. Gynaecol..

[2] Alfirevic Z., Keeney E., Dowswell T., Welton N.J., Dias S., Jones L.V., Navaratnam K., Caldwell D.M. (2015). Labour induction with prostaglandins: A systematic review and network meta-analysis. BMJ.

[3] American College of Obstetricians and Gynecologists (2009). ACOG Practice Bulletin No. 107: Induction of labor. Obstet. Gynecol..

[4] Chatsis V., Frey N. (2018). Misoprostol for Cervical Ripening and Induction of Labour: A Review of Clinical Effectiveness, Cost-Effectiveness and Guidelines.

[5] Sugimoto Y., Narumiya S. (2007). Prostaglandin E receptors. J. Biol. Chem..

[6] Narumiya S., Sugimoto Y., Ushikubi F., Alexanian A., Sorokin A., Fujii N., Singh M.S., Halili L., Boulay P., Sigal R.J. (1999). Prostanoid Receptors: Structures, Properties, and Functions. Physiol. Rev..

[7] Bakker R., Pierce S., Myers D. (2017). The role of prostaglandins E1 and E2, dinoprostone, and misoprostol in cervical ripening and the induction of labor: A mechanistic approach. Arch. Gynecol. Obstet..

[8] Austin S.C., Sanchez-Ramos L., Adair C.D. (2010). Labor induction with intravaginal misoprostol compared with the dinoprostone vaginal insert: A systematic review and metaanalysis. Am. J. Obstet. Gynecol..

[9] Young D.C., Delaney T., Armson B.A., Fanning C. (2020). Oral misoprostol, low dose vaginal misoprostol, and vaginal dinoprostone for labor induction: Randomized controlled trial. PLoS ONE.

[10] Rouzi A.A., Alsibiani S., Mansouri N., Alsinani N., Darhouse K. (2014). Randomized clinical trial between hourly titrated oral misoprostol and vaginal dinoprostone for induction of labor. Am. J. Obstet. Gynecol..

[11] Bomba-Opoń D., Drews K., Huras H., Laudański P., Paszkowski T., Wielgoś M. (2017). Polish Gynecological Society Recommendations for Labor Induction. Ginekol. Polska.

[12] Chwalisz K., Garfield R.E. (1998). Role of nitric oxide in the uterus and cervix: Implications for the management of labor. J. Périnat. Med..

[13] Ekerhovd E., Brännström M., Weijdegård B., Norström A. (2000). Nitric oxide synthases in the human cervix at term pregnancy and effects of nitric oxide on cervical smooth muscle contractility. Am. J. Obstet. Gynecol..

[14] de Vaan M.D., Ten Eikelder M.L., Jozwiak M., Palmer K.R., Davies-Tuck M., Bloemenkamp K.W., Mol B.W.J., Boulvain M. (2019). Mechanical methods for induction of labour. Cochrane Database Syst. Rev..

[15] Pierce S., Bakker R., Myers D.A., Edwards R.K. (2018). Clinical insights for cervical ripening and labor induction using prostaglandins. Am. J. Perinatol. Rep..

[16] Rugarn O., Tipping D., Powers B., Wing D. (2016). Induction of labour with retrievable prostaglandin vaginal inserts: Outcomes following retrieval due to an intrapartum adverse event. BJOG Int. J. Obstet. Gynaecol..

[17] Eikelder M.L.G.T., Rengerink K.O., Jozwiak M., de Leeuw J.W., de Graaf I.M., van Pampus M.G., Holswilder M., A Oudijk M., van Baaren G.-J., Pernet P.J.M. (2016). Induction of labour at term with oral misoprostol versus a Foley catheter (PROBAAT-II): A multicentre randomised controlled non-inferiority trial. Lancet.

[18] Papanikolaou E.G., Plachouras N., Drougia A., Andronikou S., Vlachou C., Stefos T., Paraskevaidis E., Zikopoulos K. (2004). Comparison of Misoprostol and Dinoprostone for elective induction of labour in nulliparous women at full term: A randomized prospective study. Reprod. Biol. Endocrinol..

[19] Alfirevic Z., Aflaifel N., Weeks A. (2014). Oral misoprostol for induction of labour. Cochrane Database Syst. Rev..

[20] Denguezli W., Trimech A., Haddad A., Hajjaji A., Saidani Z., Faleh R., Sakouhi M. (2007). Efficacy and safety of six hourly vaginal misoprostol versus intracervical dinoprostone: A randomized controlled trial. Arch. Gynecol. Obstet..

[21] Kerr R.S., Kumar N., Williams M.J., Cuthbert A., Aflaifel N., Haas D.M., Weeks A.D. (2021). Low-dose oral misoprostol for induction of labour. Cochrane Database Syst. Rev..

[22] Morris M., Bolnga J.W., Verave O., Aipit J., Rero A., Laman M. (2017). Safety and effectiveness of oral misoprostol for induction of labour in a resource-limited setting: A dose escalation study. BMC Pregnancy Childbirth.

[23] Nystedt A., Hildingsson I. (2014). Diverse definitions of prolonged labour and its consequences with sometimes subsequent inappropriate treatment. BMC Pregnancy Childbirth.

[24] Shetty A., Burt R., Rice P., Templeton A. (2005). Women’s perceptions, expectations and satisfaction with induced labour—A questionnaire-based study. Eur. J. Obstet. Gynecol. Reprod. Biol..

[25] Impey L. (1999). Maternal attitudes to amniotomy and labor duration: A survey in early pregnancy. Birth.

[26] Hofmeyr G.J., Gülmezoglu A.M., Pileggi C. (2010). Vaginal misoprostol for cervical ripening and induction of labour. Cochrane Database Syst. Rev..

[27] Weeks A.D., Lightly K., Mol B.W., Frohlich J., Pontefract S., Williams M.J., the Royal College of Obstetricians and Gynaecologists (2022). Evaluating misoprostol and mechanical methods for induction of labour. BJOG.

[28] Stock S.J., Ferguson E., Duffy A., Ford I., Chalmers J., Norman J. (2012). Outcomes of elective induction of labour compared with expectant management: Population based study. BMJ.

[29] Hannah M.E., Hannah W.J., Hellmann J., Hewson S., Milner R., Willan A., the Canadian Multicenter Post-term Pregnancy Trial Group (1992). Induction of Labor as Compared with Serial Antenatal Monitoring in Post-Term Pregnancy. A randomized controlled trial. The Canadian Multicenter Post-term Pregnancy Trial Group. N. Engl. J. Med..

[30] Roach V.J., Rogers M.S. (1997). Pregnancy outcome beyond 41 weeks gestation. Int. J. Gynecol. Obstet..

[31] Maggi C., Mazzoni G., Gerosa V., Fratelli N., Prefumo F., Sartori E., Lojacono A. (2019). Labor induction with misoprostol vaginal insert compared with dinoprostone vaginal insert. Acta Obstet. Gynecol. Scand..

[32] Handal-Orefice R.C., Friedman A.M., Chouinard S.M., Eke A.C., Feinberg B., Politch J., Iverson R.E., Yarrington C.D. (2019). Oral or Vaginal Misoprostol for Labor Induction and Cesarean Delivery Risk. Obstet. Gynecol..

[33] Tsikouras P., Koukouli Z., Manav B., Soilemetzidis M., Liberis A., Csorba R., Trypsianis G., Galazios G. (2016). Induction of Labor in Post-Term Nulliparous and Parous Women—Potential Advantages of Misoprostol over Dinoprostone. Geburtshilfe Frauenheilkd..

[34] Wing D.A., Brown R., Plante L.A., Miller H., Rugarn O., Powers B.L. (2013). Misoprostol vaginal insert and time to vaginal delivery: A randomized controlled trial. Obstet. Gynecol..

[35] Redling K., Schaedelin S., Huhn E.A., Hoesli I. (2018). Efficacy and safety of misoprostol vaginal insert vs. oral misoprostol for induction of labor. J. Périnat. Med..

[36] Saeed G.A., Fakhar S., Nisar N., Alam A.Y. (2011). Misoprostol for term labor induction: A randomized controlled trial. Taiwan. J. Obstet. Gynecol..

[37] Gornisiewicz T., Huras H., Kusmierska-Urban K., Galas A. (2021). Pregnancy-related comorbidities and labor induction—The effectiveness and safety of dinoprostone compared to misoprostol. Ginekol. Polska.

[38] Le Ray C., Carayol M., Bréart G., Goffinet F., for the PREMODA Study Group (2007). Elective induction of labor: Failure to follow guidelines and risk of cesarean delivery. Acta Obstet. Gynecol. Scand..

[39] Acharya T., Devkota R., Bhattarai B., Acharya R. (2017). Outcome of misoprostol and oxytocin in induction of labour. SAGE Open Med..

[40] Stephenson M.L., Powers B.L., Wing D.A. (2013). Fetal heart rate and cardiotocographic abnormalities with varying dose misoprostol vaginal inserts. J. Matern.-Fetal Neonatal Med..

[41] Kolderup L., McLean L., Grullon K., Safford K., Kilpatrick S.J. (1999). Misoprostol is more efficacious for labor induction than prostaglandin E2, but is it associated with more risk?. Am. J. Obstet. Gynecol..

[42] Kumar N., Haas D.M., Weeks A.D. (2021). Misoprostol for labour induction. Best Pract. Res. Clin. Obstet. Gynaecol..

[43] Vorherr H. (1975). Placental insufficiency in relation to postterm pregnancy and fetal postmaturity. Evaluation of fetoplacental function; management of the postterm gravida. Am. J. Obstet. Gynecol..

[44] Karcioglu O., Topacoglu H., Dikme O., Dikme O. (2018). A systematic review of the pain scales in adults: Which to use?. Am. J. Emerg. Med..

[45] Blajic I., Zagar T., Semrl N., Umek N., Lucovnik M., Pintaric T.S. (2021). Analgesic efficacy of remifentanil patient-controlled analgesia versus combined spinal-epidural technique in multiparous women during labour. Ginekol. Polska.

[46] Jodzis A., Walędziak M., Czajkowski K., Różańska-Walędziak A. (2022). Intrapartum Analgesia—Have Women’s Preferences Changed over the Last Decade?. Medicina.

[47] Czech I., Fuchs P., Fuchs A., Lorek M., Tobolska-Lorek D., Drosdzol-Cop A., Sikora J. (2018). Pharmacological and Non-Pharmacological Methods of Labour Pain Relief—Establishment of Effectiveness and Comparison. Int. J. Environ. Res. Public Health.

[48] Santana L.S., Gallo R.B.S., Ferreira C.H.J., Duarte G., Quintana S.M., Marcolin A.C. (2015). Transcutaneous electrical nerve stimulation (TENS) reduces pain and postpones the need for pharmacological analgesia during labour: A randomised trial. J. Physiother..

[49] Levett K., Smith C., Dahlen H., Bensoussan A. (2014). Acupuncture and acupressure for pain management in labour and birth: A critical narrative review of current systematic review evidence. Complement. Ther. Med..

[50] Zutshi V., Rani K.U., Marwah S., Patel M. (2016). Efficacy of intravenous infusion of acetaminophen for intrapartum analgesia. J. Clin. Diagn. Res. JCDR.

[51] Bebbington M., Pevzner L., Schmuel E., Bernstein P., Dayal A., Barnhard J., Chazotte C., Merkatz I. (2003). Uterine tachysystole and hyperstimulation during induction of labor. Am. J. Obstet. Gynecol..

[52] Schmidt M., Neophytou M., Hars O., Freudenberg J., Kühnert M. (2019). Clinical experience with misoprostol vaginal insert for induction of labor: A prospective clinical observational study. Arch. Gynecol. Obstet..

[53] Rahimi M., Haghighi L., Baradaran H.R., Azami M., Larijani S.S., Kazemzadeh P., Moradi Y. (2023). Comparison of the effect of oral and vaginal misoprostol on labor induction: Updating a systematic review and meta-analysis of interventional studies. Eur. J. Med. Res..

